# Strategies for Improving siRNA-Induced Gene Silencing Efficiency

**DOI:** 10.15171/apb.2017.072

**Published:** 2017-12-31

**Authors:** Fatemeh Safari, Solmaz Rahmani Barouji, Ali Mohammad Tamaddon

**Affiliations:** ^1^Medical Biotechnology Department, Faculty of Advanced Medical Sciences, Tabriz University of Medical Sciences, Tabriz, Iran.; ^2^Shiraz University of Medical Sciences, Shiraz, Iran.; ^3^Student Research Committee, Tabriz University of Medical Sciences, Tabriz, Iran.; ^4^Department of Traditional Medicine, Faculty of Traditional Medicine, University of Medical Sciences, Tabriz, Iran.; ^5^Center for Pharmaceutical Nanotechnology and Biomaterials, Faculty of Pharmacy, Shiraz University of Medical Sciences, Shiraz, Iran.

**Keywords:** RNA Interference, hTERT, Gene silencing

## Abstract

***Purpose:*** Human telomerase reverse transcriptase (hTERT) plays a crucial role in tumorigenesis and progression of cancers. Gene silencing of hTERT by short interfering RNA (siRNA) is considered as a promising strategy for cancer gene therapy. Various algorithms have been devised for designing a high efficient siRNA which is a significant issue in the clinical usage. Thereby, in the present study, the relation of siRNA designing criteria and the gene silencing efficiency was evaluated.

***Methods:*** The siRNA sequences were designed and characterized by using on line soft wares. Cationic co-polymer (polyethylene glycol-g-polyethylene imine (PEG-g-PEI)) was used for the construction of polyelectrolyte complexes (PECs) containing siRNAs. The cellular uptake of the PECs was evaluated. The gene silencing efficiency of different siRNA sequences was investigated and the effect of observing the rational designing on the functionality of siRNAs was assessed.

***Results:*** The size of PEG-g-PEI siRNA with N/P (Nitrogen/Phosphate) ratio of 2.5 was 114 ± 0.645 nm. The transfection efficiency of PECs was desirable (95.5% ± 2.4%.). The results of Real-Time PCR showed that main sequence (MS) reduced the hTERT expression up to 90% and control positive sequence (CPS) up to 63%. These findings demonstrated that the accessibility to the target site has priority than the other criteria such as sequence preferences and thermodynamic features.

***Conclusion:*** siRNA opens a hopeful window in cancer therapy which provides a convenient and tolerable therapeutic approach. Thereby, using the set of criteria and rational algorithms in the designing of siRNA remarkably affect the gene silencing efficiency.

## Introduction

 The hTERT (human telomerase reverse transcriptase) gene is a key component of the telomerase catalytic activity, which encodes the reverse transcriptase part of the telomerase complex.^1^ The abnormity of hTERT is considered to be associated with tumorigenesis in 85% of various tumors such as breast, prostate, lung, liver, pancreas, and colon cancer.^2^ Down-regulation of hTERT has been introduced as a highly attractive approach for cancer treatment.

 RNA interference (RNAi) is a sequence-specific gene silencing system which knocks down the gene expression by using double-stranded RNA (dsRNA) homologous to the target gene.^3^ The capability of siRNA to induce gene silencing introduces this mechanism as a powerful functional genomic tool.^4^ The gene silencing effects of RNAi can be exerted by two different mechanisms. Small interfering RNAs (siRNAs), a sequence containing 21 nucleotides (nt) with two nucleotide overhang, transiently knock down the genes.^5^ Short hairpin RNAs (shRNAs) principally provide the stable silencing of genes and are processed by Dicer into siRNAs.^6^ The gene silencing mediated by siRNA is started by the direct entrance of siRNA into the RNA induced silencing complex (RISC). The assembling of the guide (antisense) strand with the RISC, activates this complex. The RISC activation induces different mechanisms varying from the repression of translation to the degradation of mRNA which is dependent on the target site of mRNA. Targeting the coding sequence (CDS) with siRNA modulates transcript levels by Argonaute (Ago)2-mediated transcript cleavage. However, targeting 3′untranslated region (UTR) of mRNA by complementary siRNA leads to translational repression which mediated by Ago1, Ago3 and Ago4.^7,8^

 The efficiency of siRNA is dependent on various factors including sequence space, target availability, the position of nucleotides, secondary structures of mRNA and intrinsic characteristics of siRNA and target mRNA. Sequence space is the region of a gene which is selected for targeting by siRNA. Recently, regions of the gene that are transcribed such as the 5′ and 3′ UTRs and open reading frame (ORF) have been introduced as the appropriate targets. But currently, the regions about 50–100 nucleotides downstream of the start codon in the ORF is recommended. Because in the 5′UTR and sequences close to the start codon, regulatory proteins make space hindrance for RISC and interfere with silencing effect.^9^

 At the first step of siRNA designing, the target sequence is searched to find motifs such as 5′-AA(N19)TT, 5′-AA(N21) or 5′-NA(N21). The GC content of the sequence is an important parameter that affects siRNA functionality. It is recommended to choose the sequences with the low to the medium proportion (30–64%) of GC content.^10^

 The propensity of a duplex to form internal hairpins and the relative stability are two significant factors of siRNA efficiency which can be estimated by prediction of melting temperatures (Tm).^11^ Sequences with the Tm of 20-60 °C is recommended because the siRNAs with this range of Tm are better silencer.^12^ Another strong determinant of siRNA functionality is the secondary structures of the mRNA which represents the level of the accessibility to the target site.^13^ The asymmetry of siRNA duplex ends is a significant parameter for improvement of siRNA efficacy. This parameter is based on the requirement of less stable 5′end of the antisense strand than 5′end of the sense strand. Achieving this asymmetry needs high A/U content at the 5′end of the antisense strand and high G/C at the 5′end of the sense strand.^14,15^ Also, a number of position-specific nucleotide preferences have an influence on the gene silencing efficiency of siRNA (Table 1).^16^ The ability of sense and antisense strands to form the duplex has a direct relation with a functionality of siRNA, so any secondary structures may diminish the functionality.

 In addition, checking the specificity of siRNA is a crucial issue to reduce the risk of unintended gene silencing which is called off-target effect. Both strands of siRNA must be checked by the BLAST (basic local alignment search tool) online software. The siRNA sequence represents less than 78% query coverage with other genes may be a desirable sequence.^17^

**Table 1:** A combination of the most important criteria for siRNA designing and scoring system

**Criteria**	**score**
GC content 30-52%	1
Up to 5 A/U in position 15-19 of S strand	1 per A/U
Inverse repeat	1
A in position 19	1
A in position 3	1
U in position 10	1
G/C in position 19	1
G in position 13	-1
Relative stability of internal siRNA duplex	1
Internal stability	1

 Considering these issues, various rational algorithms have been devised for designing more efficient siRNAs.^15,16,18^ Each of these algorithms has focused on the specific dimensions but, sometimes overlap with each other. On the basis of these algorithms, lots of online soft wares have emerged in the area of siRNA designing. Each of these computational tools is in accordance with different parameters to provide an individual scoring system for siRNA designing (Table 2).

 Numerous studies have investigated the effects of the rational designing on the gene silencing activity of siRNA. In the study ahead, the effect of these criteria and their priority on the efficiency of siRNA were assessed. Our findings showed that the accessibility to the target site is more important than other criteria. However, in designing an efficient siRNA other parameters such as thermodynamic features and sequence preferences should be observed.

**Table 2:** Names and addresses of siRNA design computational soft wares

**Soft wares**	**Address**
siDirect	http://genomics.jp/sidirect/index.php?type=fc
siDESIGN Center	dharmacon.gelifesciences.com/design-center
siRNA Design Software	http://www.genscript.com/ssl-bin/app/rnai
Block-iT RNAi Designer	https://rnaidesigner.invitrogen.com
siRNA Target Finder	http://www.ambion.com/techlib/misc/siRNA_finder.html
RNAi explorer	www.genelink.com/sirna/siRNAorder.asp

## Materials and Methods

 Poly (ethylene glycol)-g-polyethylenimine (PEG-g-PEI (PEG; 5 kDa and PEI; 25 kDa)) was synthesized by the pharmaceutics department of the Pharmacy School, University of Medical Sciences (Shiraz, Iran). Human lung adenocarcinoma cell line (A549) was obtained from Pasture Institute (Tehran, Iran). siRNA against hTERT, scramble siRNA, and positive control siRNA sequences were synthesized by Qiagen (Korea). Real-time PCR Kit was purchased from TaKaRa Biotechnology, (Dalian, China). Cell culture medium and fetal bovine serum (FBS) were purchased from GIBCO (Carlsbad, CA, USA).

###  The in-silico protocol for siRNA designing 

 The accession number of hTERT (AB085628) was extracted from the national center for biotechnology information (NCBI). Ambion® software (www.lifetechnologies.com) was used for designing of siRNA which provided a list of candidate siRNAs. In the selection, 5´ UTR, 3´ UTR, start codon, introns and splice junctions were avoided.

 Analyzing the internal instability, the asymmetry of siRNA duplex and secondary structures of candidate siRNAs were accomplished by Sfold software (http://sfold.wadsworth.org). Melting temperature (Tm) of the siRNA hairpin loop is another thermodynamic feature of siRNA which was predicted by "OligoAnalyzer 3.1" online service (https://eu.idtdna.com/calc/analyzer). BLAST (http://blast.ncbi.nlm.nih.gov) as a worldwide algorithm was used to evaluate the off-target effects. In accordance with the output of soft wares, main sequence siRNA (MS) was selected and synthesized. To confirm the functionality of MS, scramble sequence (SS) as a control negative was designed. Furthermore, the control positive sequence (CPS) was selected from the literature. The sequences of these three siRNAs are listed in Table 3.

**Table 3:** Sequence of siRNAs. MS: main sequence, CPS: control positive sequence, SS: scramble sequence

**Sequences name**	**strand**	**Molecular weight**	**sequences**
MS	Sense	13315	GCACUUCCUCUACUCCUCATT
MS	Antisense	13315	UGAGGAGUAGAGGAAGUGCTT
CPS	Sense	13315	UGAUUUCUUGUUGGUGACATT
CPS	Antisense	13315	UGUCACCAACAAGAAAUCATT
SS	Sense	13315	UGAUUUCUUGUUGGUGACATT
SS	Antisense	13315	UGUCACCAACAAGAAAUCATT

###  Cell Culture 

 Human lung adenocarcinoma A549 cells were cultured and maintained in RPMI 1641 medium containing 10% fetal bovine serum and antibiotics at 37°C in a humidified atmosphere of 5% CO2.

## Synthesis and characterization of PEG-g-PEI nanoparticles for siRNA delivery

 The shortcomings of siRNA such as the lack of in vivo stability and the poor permeability of cell membranes propelled the researchers to devise efficient carriers. To this end, PEG-g-PEI nanoparticles have been introduced as an effective tool for siRNA delivery. Therefore, in this study PEG-g-PEI nanoparticles were used as a transfection agent. PEG-g-PEI nanoparticles were synthesized and characterized as previously described.^19^

###  Preparation of Polyelectrolyte Complex (PECs) 

 PECs containing hTERT siRNA was prepared by adding appropriate volumes of siRNA (20µM) and PEG-g-PEI solution to deionized water. The mixture was gently blended and incubated for 20 minutes at room temperature.^20^ Relay on previous data, N/P (Nitrogen/Phosphate) ratio of 2.5 was chosen as an appropriate ratio for PECs formation.^19^

###  PECs characterization 

 The surface charge and diameter of the PECs were determined by measuring of zeta potential and dynamic light scattering (Brookhaven, NY, USA) as previously described.^21^ The ability of PEG-g-PEI to condense siRNA into nanoparticles was analyzed by ethidium bromide dye-exclusion assay. The relative stability of PECs was evaluated by measuring the release of siRNA from nanoparticles in the presence of a heparin sulfate.

###  Transfection Efficiency Assay

 Cells (A549 cell line) were seeded in 96 well cell culture plates (1×10^4^ cells/well) and incubated for 24 hours prior to treatment. PECs containing FITC-labeled PEG-g-PEI/siRNA and FITC-labeled PEI/siRNA at the N/P ratios of 2.5 were prepared in RPMI. Cell culture media (100 μL) with the final concentration of 200 nM siRNA were added to each well. After 4h incubation, on the basis of previous protocol^20^ samples were fixed and the cellular uptake of PECs were assessed by flow cytometry. Finally, data acquisition and analysis was performed by recruiting WinMDI software.

###  Quantitative Real-time PCR 

 The attenuation of gene expression mediated by hTERT siRNA was analyzed by quantitative Real-Time PCR. Thereby, 48h after PECs transfection; total RNA was extracted by using TriPure isolation reagent (Invitrogen, Carlsbad, CA, USA) according to the manufacturer’s instructions. Reverse transcription and Real-time PCR were performed based on the protocol of SYBR Green-I _ RT-PCR Kit (TaKaRa, China). The list of primers which were used for Real-time PCR is represented in Table 4.

**Table 4:** Sequence of the primers used in Real Time PCR

**Oligonucleotides**		**Primer sequences**	**Position**	**PCR product length**
**hTERT**	Forward	5′ CCGCCTGAGCTGTACTTGT 3′	2156F	198
	Reverse	5' CAGGTGAGCCACGAACTGT 3'	2362R	
**Beta-actin**	Forward	5′ TCCCTGGAGAAGAGCTACG3′	787F	131
	Reverse	5′GTAGTTTCGTGGATGCCACA3′	917R	

###  Statistical Analysis Synthesis of and characterization of PEG-g-PEI nanoparticles

 Data were expressed as a mean ± standard error (SE). All statistical analyses were performed with SPSS 11.0 software. Analysis of variance (ANOVA) was used, and a P-value ≤ 0.05 was considered as significant.

## Results

###  Characterization of siRNAs

**Figure 1:**
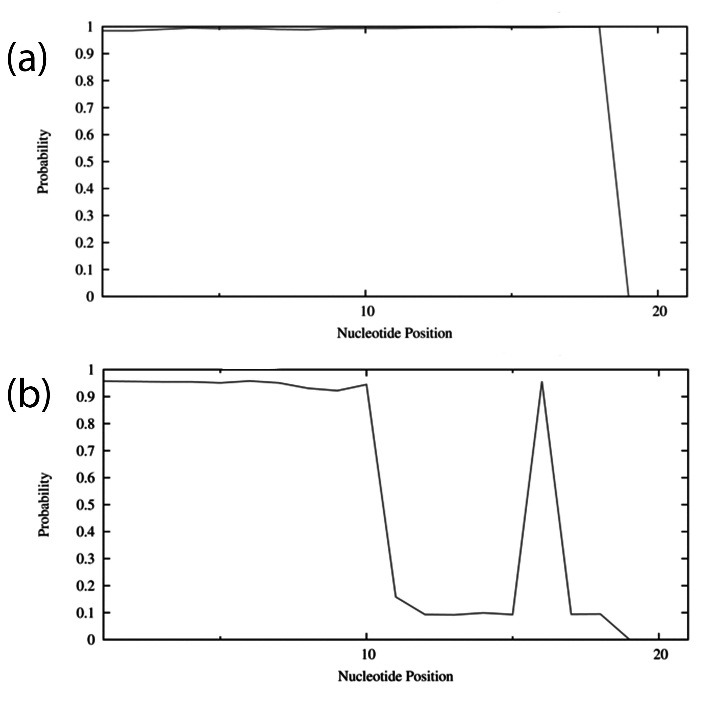
The accessibility to the target site for each nucleotide in the sequence. a: the target accessibility of main sequence (MS). b: the target accessibility of control positive sequence (CPS).

 Characters of siRNA sequences including target accessibility score, duplex feature score, duplex thermodynamics score, GC content and, etc were investigated with Sfold online tool. The results of the analysis are shown in Table 5. The Tm of CPS was 38.4°C and MS was 50.4 °C which were in the appropriate ranges. Also, the target accessibility of both MS and CPS sequences are represented in Figure 1. The results of BLAST showed that MS had 100% similarity with hTERT gene and less than 78% homology with the other part of the genome.

**Table 5:** Individual scores for duplex features and thermodynamic

**Duplex features and thermodynamic**	**Main sequence (MS)**	**Control positive sequence (CPS)**
GC content	0	1
A/U in position 15	0	1
A/U in position 16	0	0
A/U in position 17	1	1
A/U in position 18	0	0
A/U in position 19	1	1
Inverse repeat	1	1
A in position 19	1	1
A in position 3	1	1
U in position 10	0	0
G/C in position 19	0	0
G in position 13	0	-1
Relative stability of internal siRNA duplex	1	0
Internal stability	0	1

###  PECs characterization 

 The size and zeta potential were determined at PEG grafting degree of 0.2 and N/P ratio of 2.5. Size of this nanoparticle was 114 ± 0.645 nm. PEG grafting degree of 0.2 represented negative zeta potential due to charge covering effect of PEG grafting. The findings of ethidium bromide dye-exclusion assay showed that the complexation of siRNA and PEG-g-PEI resulted in decreasing in the intercalating property of ethidium bromide.^19^ The results of polyanion competition assays demonstrated that PEG grafting improved the stability of the PECs.^19^

###  In vitro Transfection Assay

**Figure 2:**
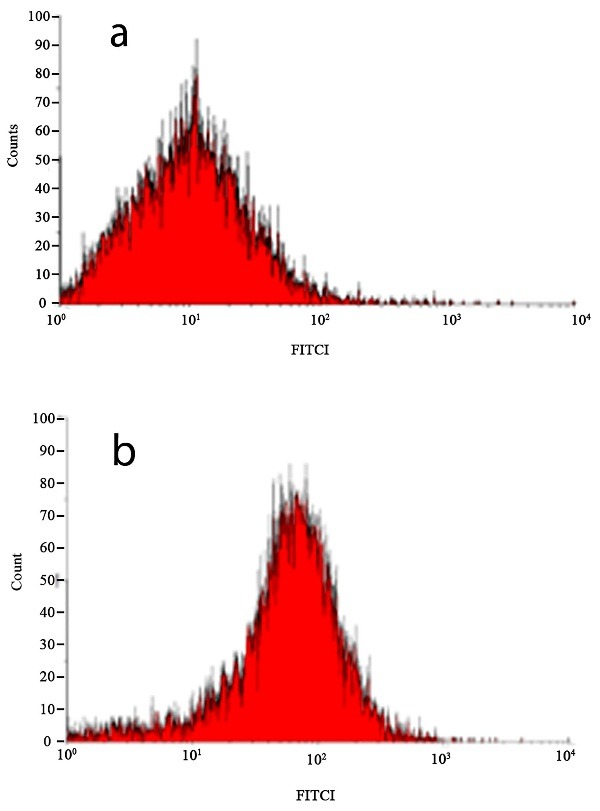
FACS histogram of FITC positive cells after FITC-labeled siRNA transfection. a: PEI/FITC labeled siRNA transfected cells. b: PEG-g-PEI/FITC-labeled siRNA transfected cells.

 The transfection efficiency of the complexes was evaluated with a flow cytometry. FITC-labeled siRNAs were used to form these complexes. FACs results showed the transfection efficiency of PEI/siRNA was 81 ±2.5 and PEG-g-PEI/siRNA was 95.5% ± 2.4%. PEI/siRNA was used as a control positive. The histogram plots of PEG-g-PEI/siRNA and PEI/siRNA transfection efficiency are represented in Figure 2. Shifting of plot to right side showed that the transfection efficiency of PEG-g-PEI/siRNA is improved in comparison with PEI/siRNA.

###  The Gene Silencing Effect of PECs

**Figure 3:**
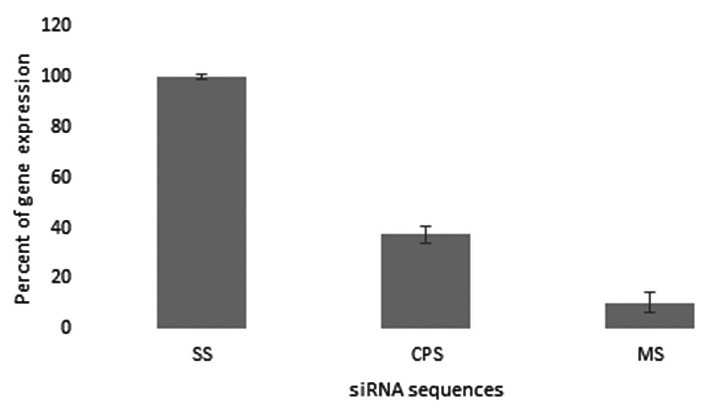
The results of quantitative real-time PCR. (main sequence [MS], control, positive sequence [CPS] and scramble sequence [SS].

 Real-time PCR was used to evaluate the potency of MS and CPS to reduce the hTERT mRNA levels respectively. Both MS and CPS reduced the expression of hTERT by targeting its complimentary mRNA. However, attenuation induced by MS was much more than CPS. No obvious changes in hTERT expression of SS, confirmed the results of the other sequences (2−ΔΔCT= 1.27) (Figure 3).

## Discussion

 RNAi-related strategies have become promising methods in various fields of research. Effective gene silencing by the RNAi pathway needs a comprehensive understanding of the criteria that affect siRNA functionality and specificity. Target site, nucleotide content, the accessibility of the target site and thermodynamic features are criteria that influence the functionality of siRNA. Recent experiences showed that siRNAs closer to the start codon are more efficient than farther ones.^22^ Based on the algorithm by Reynolds et al., the sequence preferences of the sense strand are influential on the siRNA efficiency. Reynolds studies on the sense strand showed that the presence of A at the third position and U at the tenth position, A at the nineteenth position, besides the absence of G or C at the nineteenth and G at the thirteenth nucleotide potentiate the silencing effect of siRNA.^16^ According to Jagla and his research team analysis the presence of at least 3 A/U at position 13–19 increases the efficiency of predicted siRNA.^23^ In addition, low internal stability at 5′-end of the antisense strand is required for assembling of the RISC complex.^24^ The number of A/U at 5′-end of the antisense strand determines the internal stability.^25^ So in the 5′-end of antisense strand, four out of the seven nucleotides must be A or U.^26^

 Tm as a thermodynamic parameter affects the functionality of siRNA so that high Tm values give rise to internal hairpin structures and low values lead to high internal repeat stability. Sorting the functional siRNA by Tm revealed that duplexes lacking internal hairpin structures and stable internal repeats were better silencers.

 GC content is an important parameter in the efficiency of siRNAs because low GC content gives rise to weak and unspecific binding, while high GC content may impede unwinding the siRNA duplex by RISC complex and helicase. To this end, the different ranges of GC content were suggested by different algorithms such as 31.6-57.9%^18^ and 36–52%.^16^ Computational modeling suggested that target secondary structure and accessibility are two influential factors which affect the potency of siRNA.^27,28^ The target accessibility is necessary for the downstream step of target recognition for gene silencing mediated by RNAi. The importance of target accessibility for RNAi pathway has been approved by the study of HIV. A single point mutation alters the accessibility to the target site and HIV-1 can escape from RNAi pathway.^29^ However, the target accessibility is a crucial parameter for functional siRNA but duplex asymmetry is also important and both of them together can greatly improve the efficiency of RNAi.^30^

 In this study, the analysis of sequences by OligoAnalyzer and Sfold soft wares showed that nucleotide preferences were more observed in CPS than MS. The GC content of MS was 52.6% and CPS was 36.8%. According to these results, the CPS must be more efficient than MS, but findings of Real-time PCR demonstrated that the gene silencing efficiency of MS (up to 90%) was more than CPS (up to 63%). This controversy is due to the higher target accessibility of MS than CPS which was represented in Figure 1. The local structure of mRNA at the target site determines the level of accessibility of siRNA to target mRNA. The findings of SHAO and his co-workers suggested that, after RISC assembly, the secondary structure of target mRNA plays a crucial role in target binding by the guide of the antisense strand. Thus, to achieve effective silencing by RNAi, the selected siRNAs must have sequence features that facilitate RISC activation, as well as target accessibility that improves target recognition by intermolecular base-pairing.^30^

 Poor cellular uptake and hydrolytic sensitivity of naked polyanionic oligonucleotides are the main concerns in current nucleic acid-based therapeutic strategies which lead the researchers to find the efficient and safe delivery systems.^31^ Cationic polymers and block copolymers are polymeric condensing carriers for gene delivery due to good stability, simple preparation and easily modified chemical structures.^32^ PEI, as a branched or linear cationic polymer, has been used in siRNA delivery because of low immunogenic stimulation and safety issues. In this study, PEI was used as nano-carrier for gene delivery, But to improve the biocompatibility and transfection efficiency, the PEI core was grafted to hydrophilic PEG. In the characterization of PECs, ethidium bromide dye exclusion assay showed that condensation of siRNA by PEG-g-PEI inhibited the intercalating property of ethidium bromide in the siRNA backbone. In addition, heparin competition assay demonstrated that PEG grafting improves the stability of the PECs.^19^ The results of transfection efficiency showed that PEG grafting increases the cellular uptake of PEG-g-PEI/siRNA in comparison with PEI/siRNA which is related to the improving of PECs stability.

## Conclusion

 siRNA-based gene therapies are emerging as a promising novel therapeutic approach for the treatment of various diseases such as cancer. More than 20 RNAi therapeutic agents are currently in clinical trials. Several of these clinically relevant RNAi therapies are in Phase III which represents that the first RNAi therapeutic to be clinically is not so far. Therefore, considering to design efficient and specific siRNAs for clinical uses is so crucial. To this end, various criteria and algorithms have been devised for achieving high functional siRNA. Some of these criteria have the priority than the other so observing these criteria may greatly affect the clinical efficiency of siRNA. However, novel genome editing technologies such as CRISPR revolutionize the gene therapy approaches and comes to the clinic for treatment of the broad spectrum of diseases.

## Ethical Issues

 Not applicable

## Conflict of Interest

 The authors declare no conflict of interests.
